# Pathogenic influenza B virus in the ferret model establishes lower respiratory tract infection

**DOI:** 10.1099/vir.0.064352-0

**Published:** 2014-10

**Authors:** Stephen S. H. Huang, David Banner, Stephane G. Paquette, Alberto J. Leon, Alyson A. Kelvin, David J. Kelvin

**Affiliations:** 1 Division of Experimental Therapeutics, Toronto General Research Institute, University Health Network, Toronto, ON, Canada; 2 Department of Immunology, Faculty of Medicine, University of Toronto, Toronto, ON, Canada; 3 Institute of Medical Science, Faculty of Medicine, University of Toronto, Toronto, ON, Canada; 4 Immune Diagnostics & Research, Toronto, ON, Canada; 5 International Institute of Infection and Immunity, Shantou University Medical College, Shantou, Guangdong, PR China; 6 Dipartimento di Scienze Biomediche, Universita' degli Studi di Sassari, Sassari, Sardinia, Italy

## Abstract

Influenza B viruses have become increasingly more prominent during influenza seasons. Influenza B infection is typically considered a mild disease and receives less attention than influenza A, but has been causing 20 to 50 % of the total influenza incidence in several regions around the world. Although there is increasing evidence of mid to lower respiratory tract diseases such as bronchitis and pneumonia in influenza B patients, little is known about the pathogenesis of recent influenza B viruses. Here we investigated the clinical and pathological profiles of infection with strains representing the two current co-circulating B lineages (B/Yamagata and B/Victoria) in the ferret model. Specifically, we studied two B/Victoria (B/Brisbane/60/2008 and B/Bolivia/1526/2010) and two B/Yamagata (B/Florida/04/2006 and B/Wisconsin/01/2010) strain infections in ferrets and observed strain-specific but not lineage-specific pathogenicity. We found B/Brisbane/60/2008 caused the most severe clinical illness and B/Brisbane/60/2008 and the B/Yamagata strains instigated pathology in the middle to lower respiratory tract. Importantly, B/Brisbane/60/2008 established efficient lower respiratory tract infection with high viral burden. Our phylogenetic analyses demonstrate profound reassortment among recent influenza B viruses, which indicates the genetic make-up of B/Brisbane/60/2008 differs from the other strains. This may explain the pathogenicity difference post-infection in ferrets.

## Introduction

The influenza virus is a constant health concern. Influenza B infections average between 20–30 % of total influenza incidence but can be 50 % or greater depending on the season (Paul Glezen *et al.*, 2013). Two influenza B lineages, B/Yamagata/16/88 (B/Yam) and B/Victoria/2/87 (B/Vic), have been co-circulating since the 2009 H1N1 pandemic (WHO, 2014).

There are also significant differences in the evolution and epidemiology between influenza A and B viruses. Influenza A infects a broad range of natural vertebrate hosts which gives rise to various subtypes whereas humans are the primary host of influenza B viruses (Ohishi *et al.*, 2002; Osterhaus *et al.*, 2000). For influenza B viruses, a narrow host range is thought to limit subtype diversity and gene reassortment; furthermore, the rate of antigenic drift has been estimated to be slower than for A viruses (Chen & Holmes, 2008; Lindstrom *et al.*, 1999; McCullers *et al.*, 1999). As a result, influenza B viruses are thought to evolve slower than influenza A viruses; there has been no evidence of influenza pandemic caused by a B virus (Cheng *et al.*, 2012; Hay *et al.*, 2001; Paul Glezen *et al.*, 2013).

Influenza B viruses are often considered to cause mild clinical disease predominantly in paediatric and geriatric patients (Chan *et al.*, 2013; Chi *et al.*, 2008; Li *et al.*, 2008; Wang *et al.*, 2008). Despite this, several lines of evidence exist suggesting these viruses are also capable of causing significant human disease. Comparative human clinical reports suggest that influenza B infection may induce severe clinical and inflammatory responses comparable to influenza A (Kaji *et al.*, 2003; Sakudo *et al.*, 2012). Influenza B virus dominant seasons have also been documented such as the 2002–2003 season when the prevalence of influenza B infection was higher than that of influenza A infection in most countries around the world.

Ferrets are a suitable model to study influenza infection as they manifest human-like flu symptoms and immune responses (Banner & Kelvin, 2012; Hamelin *et al.*, 2010; Hause *et al.*, 2013; Huang *et al.*, 2013; León *et al.*, 2013). Previously, we found distinct clinical and immunological patterns between ferrets infected with B/Vic and B/Yam lineages, where B/Vic appeared to be the more virulent lineage (Huang *et al.*, 2011). Here we investigated influenza B virus infection in ferrets utilizing four unique strains, two B/Yam (B/Florida/04/2006 and B/Wisconsin/01/2010) and two B/Vic viruses (B/Brisbane/60/2008 and B/Bolivia/1526/2010), that are representative of circulating B viruses. We then analysed clinical characteristics, viral kinetics and histopathology throughout the respiratory tract. Phylogenetic analysis of representative influenza B strains from the past 30 years was employed to understand the genetic relationship of these viruses. These findings will help to characterize the clinical severity of influenza B and establish the ferret influenza B model. This work provides insight into the development of appropriate influenza therapeutic strategies.

## Results

### Distinct clinical features among influenza B infections in ferrets

A clear clinical and pathological picture of recent B/Yam and B/Vic lineage infections has not yet, to our knowledge, been described. Here we studied the pathogenicity of B viruses to enhance the understanding of disease progression and strain variation. We infected healthy male naive adult ferrets with either B/Florida/04/2006 (B/Fla), B/Wisconsin/01/2010 (B/Wisc), B/Brisbane/60/2008 (B/Bris) or B/Bolivia/1526/2010 (B/Bol). We monitored daily temperature and weight changes, nasal symptoms, sneezing and inactivity level for 7 days post-infection (pi).

**Fig. 1.**
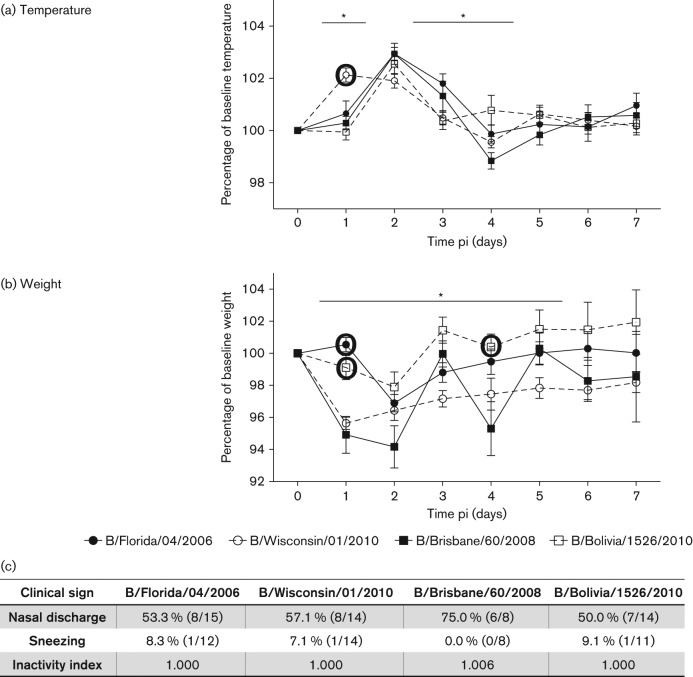
B/Brisbane/60/2008 infection induces a severe clinical outcome. Daily clinical signs of adult ferrets infected with B/Brisbane/60/2008 (*n* = 8), B/Florida/04/2006 (*n* = 15), B/Wisconsin/01/2010 (*n* = 14) or B/Bolivia/1526/2010 (*n* = 14) at 10^6^ EID_50_ (50 % egg infectious dose) were measured over a 7 day time-course. Body temperature (a) and weight (b) are expressed as a percentage baseline values. Percentage nasal discharge and sneezing were observed and calculated daily, and physical inactivity index (baseline of 1.000) of each infection is shown (c). Error bars represent sem. *p<0.05 and large bold circles represent significant difference from B/Brisbane/60/2008 infection by Bonferroni–Holm test (post-hoc analysis).

Generally, influenza B infected animals experienced fever of a 2 day duration peaking on day 2 pi (except B/Wisc) (Fig. 1a). No significant difference was found between the temperature peak values. Conversely, kinetics of weight change were more distinct among infections (Fig. 1b). B/Wisc, B/Fla and B/Bol infected animals experienced weight loss (peak values: 4.5, 3.1 and 2.1 %, respectively) for 1 or 2 days pi then began recovery. In contrast, B/Bris infected animals had a biphasic weight loss curve and began to lose weight 1 day pi, peaking on day 2 pi (6 %), then experienced a secondary weight loss on day 4 pi (4 %). Nasal discharge was prominent among all infections, with the highest amount found in B/Bris infected ferrets (6/8 animals) (Fig. 1c). Sneezing was not readily observed and the activity level was not affected. B/Bris infected animals displayed minimal inactivity (not significantly different from the other viruses). The time-courses pi of nasal discharge, sneezing and inactivity level are included in Fig. S1 (available in the online Supplementary Material). Nasal discharge peaked 2 to 3 days pi, which followed the fever kinetics (1 to 3 days pi). Impact on the activity of the B/Bris infected ferrets was observed on day 3 pi, which followed fever and preceded the second peak weight loss. No prominent sneezing was observed in this study. In summary, we found B/Bris virus (B/Vic) gave the highest morbidity (most weight loss and longest duration) while B/Bol (B/Vic) induced the mildest clinical illness, suggesting strains from the same lineages do not necessarily impose a similar host clinical impact.

### B/Bris virus replicated in the ferret lower respiratory tract

Severe influenza disease has been associated with the development of lower respiratory tract infection (Taubenberger & Morens, 2008). Since ferrets and humans have similar distribution of influenza receptors in their respiratory tracts (Jayaraman *et al.*, 2012; Maher & DeStefano, 2004; Suzuki *et al.*, 2000; van Riel *et al.*, 2007), ferrets are a suitable model to evaluate human influenza replication *in vivo*. Here, we measured viral titres in nasal wash, trachea and lung to represent viral burden in the upper, middle and lower respiratory tract during influenza B virus infection.

**Fig. 2.**
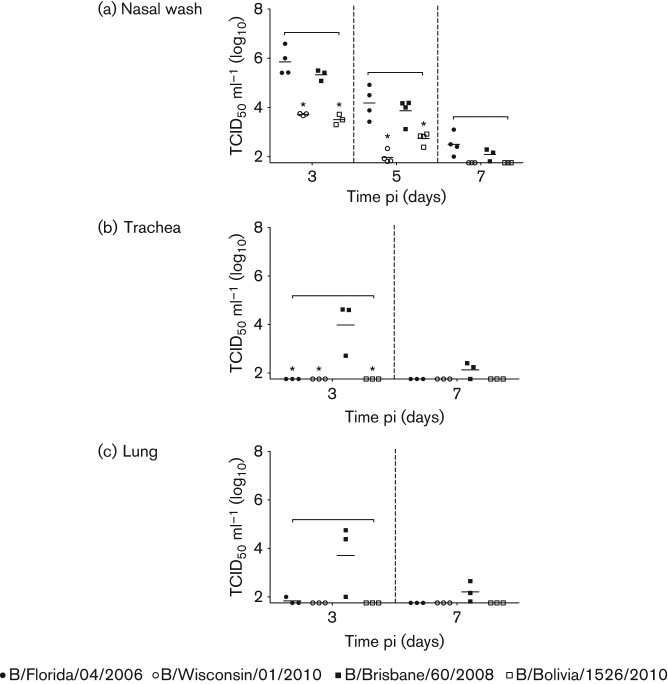
Influenza B viruses replicated efficiently but were not restrictived to the upper respiratory tract. All nasal wash (a), trachea (b) and lung (c) samples from infected ferrets were collected on the specified days pi and processed before titration on MDCK cells (TCID_50_ ml^−1^). A square bracket represents significant difference among the infections (*P* <0.05) and an asterisk represents significant difference from B/Brisbane/60/2008 infection by Bonferroni–Holm test (post-hoc analysis).

From the titre kinetics in nasal washes, all influenza B viruses readily replicated in the nose, although analyses of the middle and lower respiratory tract found that only B/Bris virus was able to significantly infect the lower reaches of the respiratory tract (Fig. 2). Besides replication location, there was also a difference in virus levels. Nasal wash viral titres of B/Bris were significantly higher than the titres of B/Wisc and B/Bol on day 3 (40- and 70-fold higher than B/Wisc and B/Bol, respectively) and day 5 pi (80- and 10-fold higher than B/Wisc and B/Bol, respectively) (Fig. 2a). The virus levels between B/Bris and B/Fla were not significantly different. Importantly, B/Bris trachea and lung virus levels were markedly high and the other influenza B infections were not detectable on either day 3 or 7 pi (Fig. 2b, c). A similar trend was also observed when using real-time PCR assays, where B/Bris had the highest viral RNA (vRNA) copy number 3 days pi, although slight vRNA levels were also detected in B/Fla and B/Wisc (Fig. S2). vRNA was not detected in the B/Bol samples. These results suggested that while influenza B viruses were mainly found to be an upper respiratory tract pathogen in ferrets, there were differences in replication efficiency in the upper respiratory tract, and B/Bris replicated efficiently in the lower respiratory tract.

### Influenza B infection induced strain-specific lung pathology

Our results from viral titre kinetics demonstrated that influenza B viruses were not confined to the upper respiratory tract. Our previous study with ferrets demonstrated the B/Yam lineage was able to induce pathology in the bronchioles (Huang *et al.*, 2011); here we expanded our histopathological studies.

**Fig. 3.**
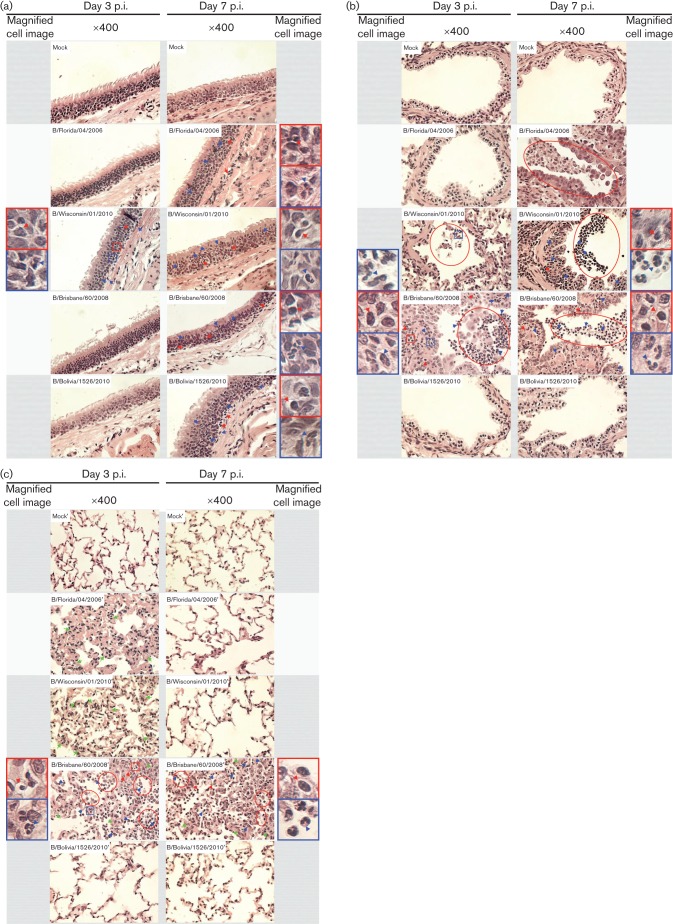
Acute onset and deteriorating pathology in the respiratory tract upon influenza B infection. Haematoxylin and eosin staining of paraffin embedded tracheas (a), bronchioles (b) and alveoli (c) from mock- or influenza B virus infected adult ferrets (day 3 or 7 pi). Red ellipses enclose areas of bronchiolar exudate with inflammatory and epithelial cells. Green arrows indicate thickened alveolar walls. Blue arrows indicate infiltrating neutrophils. Red arrows indicate cell infiltration by other leukocytes. The representative neutrophil and mononuclear leukocyte are enclosed by the blue and red boxes respectively and magnified (×400) in the image panels beside each corresponding micrograph.

In the trachea and the bronchioles, B/Wisc, and B/Wisc and B/Bris (respectively) infection induced the earliest (3 days pi) neutrophil and other mononuclear leukocyte infiltration [blue and red arrows, respectively; Fig. 3a, b (left panels)] into the epithelium. Pathology in the trachea increased by day 7 pi, when all four viruses induced leukocyte infiltration to the epithelia [neutrophils and mononuclear cells, blue and red arrows, respectively; Fig. 3a (right panel)]. However, no epithelial desquamation was observed in the trachea. Shedding of bronchiolar epithelial cells into the lumen with accompanying cellular necrosis (Fig. 3b, red ellipses) was observed and most profound in B/Bris infection. In addition, a high number of neutrophils (Fig. 3b, blue arrows) was also found within the epithelial debris of the lumen and infiltrated the remaining columnar epithelial cells in the bronchioles of B/Bris infected ferrets. Conversely, the bronchioles in the B/Fla and B/Bol infected animals (3 days pi) appeared similar to the mock-infected control. By day 7 pi, epithelial desquamation (red ellipses) and leukocyte infiltration (blue and red arrows) in the bronchioles of B/Wisc and B/Bris infected animals were still observed (Fig. 3b, right panel). Furthermore, epithelial cell sloughing was also noted in B/Fla infection by day 7 pi (red ellipse). Conversely, the bronchioles of the B/Bol infected animals appeared normal and similar to the mock-infected animals.

We also investigated the lowest end of the respiratory tract in ferrets for evidence of pathology. We observed early (3 days pi) small airway histological alteration in B/Fla, B/Wisc and B/Bris infected animals (Fig. 3c, left panel). Both B/Fla and B/Wisc infection caused mainly multifocal interstitial pneumonia determined by the thickened lining of the alveolar wall (Fig. 3c, green arrows). B/Bris infection, however, not only caused interstitial pneumonia (Fig. 3c, green arrows) by day 3 pi but also induced neutrophil and mononuclear cell infiltration to the alveolar lining and lumens (blue and red arrows respectively) and epithelial shedding (red ellipses). The severity in the alveoli had improved by day 7 pi for all strains except the B/Bris infection (Fig. 3c, right panel). The alveoli in the B/Fla and B/Wisc infected animals appeared normal and similar to the mock-infected control animals. Nevertheless, interstitial pneumonia (Fig. 3c, green arrows), cell infiltration (blue and red arrows), and epithelial shedding (red ellipses) were still observed in the alveoli of B/Bris infected animals. Alveoli of the lungs of B/Bol infected animals did not incur pathological changes throughout the time-course. Together, the four influenza B strains imposed differential pathology where B/Bris infection gave the most severe and prolonged impact, which included the lower reaches of the respiratory system while B/Bol virus induced the least damage.

### Viral segments layout in B/Bris, B/Bol, B/Flo and B/Wis

**Fig. 4.**
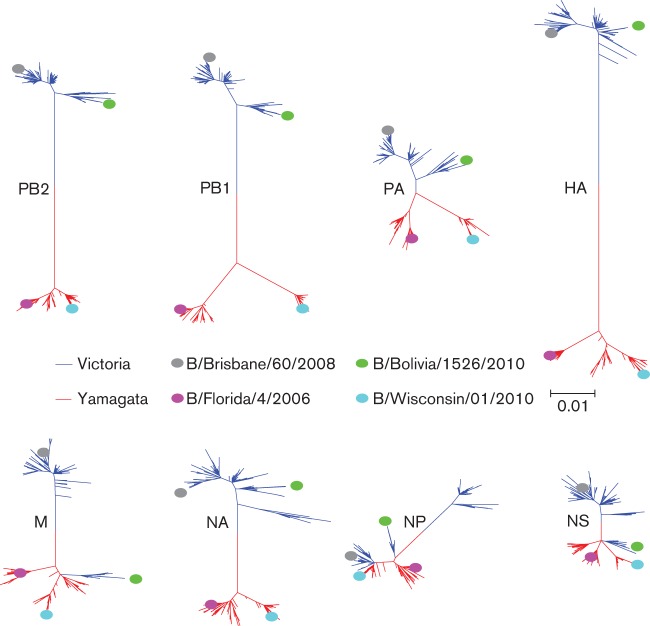
Genetic make-up of influenza B strains that circulated during 2006 and 2010. The genomic sequences of 660 strains isolated between 2006 and 2010 were downloaded from the GISAID EpiFlu database, and, together with the sequences from B/Bris, B/Bol, B/Flo and B/Wis, were subjected to maximum-likelihood clustering based on the general time-reversible model with 500 bootstrap replications using mega v.6.0.2 software. Branch lengths are proportional to number of substitutions per site. PB2, Polymerase Basic 2; PB1, Polymerase Basic 1; PA, Polymerase; M, Matrix Protein; NA, Neuraminidase; NP, Nucleoprotein.

From above, influenza B infection appeared to trigger a distinct strain-specific disease pattern and severity. Therefore, we hypothesized that each strain was composed of a unique set of gene segments leading to variability in clinical outcomes. To characterize the genetic make-up of the four influenza B strains within the context of the pool of circulating influenza B viruses, the sequences from the genomic segments of B/Bris, B/Bol, B/Flo and B/Wis, together with the sequences of 660 influenza B strains that were isolated between 2006 and 2010, were grouped by maximum-likelihood clustering (Fig. 4). The grouping patterns of the PB2, PB1, PA, HA and NA gene segments revealed that B/Bris, B/Bol, B/Flo and B/Wis belong to four different groups of strains that circulated between 2006 and 2010 and which evolved independently. The NP sequences of these four strains also cluster within four independent groups, however, an additional group within the Victoria lineage that presents differentiated NP genes is also present. Interestingly, a group formed by B/Bol and other members of the Victoria lineage incorporated Yamagata-derived M and NS genes; as a result, only B/Bris remained within the group of most ‘pure’ Victoria strains. Additional *in vitro* and *in vivo* studies are required to determine whether the presence of Yamagata-derived M and NS proteins in B/Bol may have influenced its pathogenic features.

### Phylogenetic analyses of influenza B viruses show gene rearrangement between lineages

**Fig. 5.**
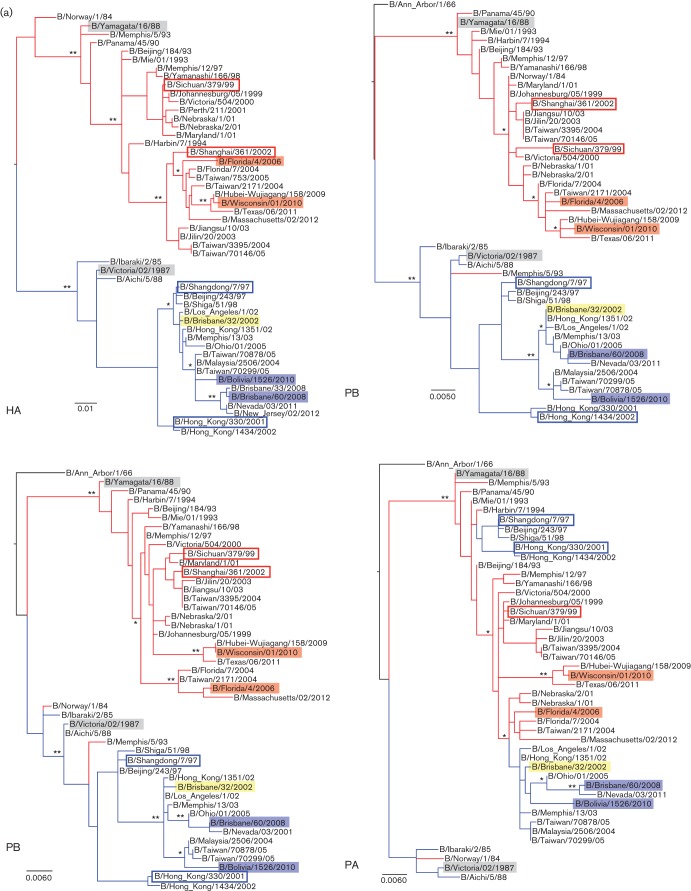

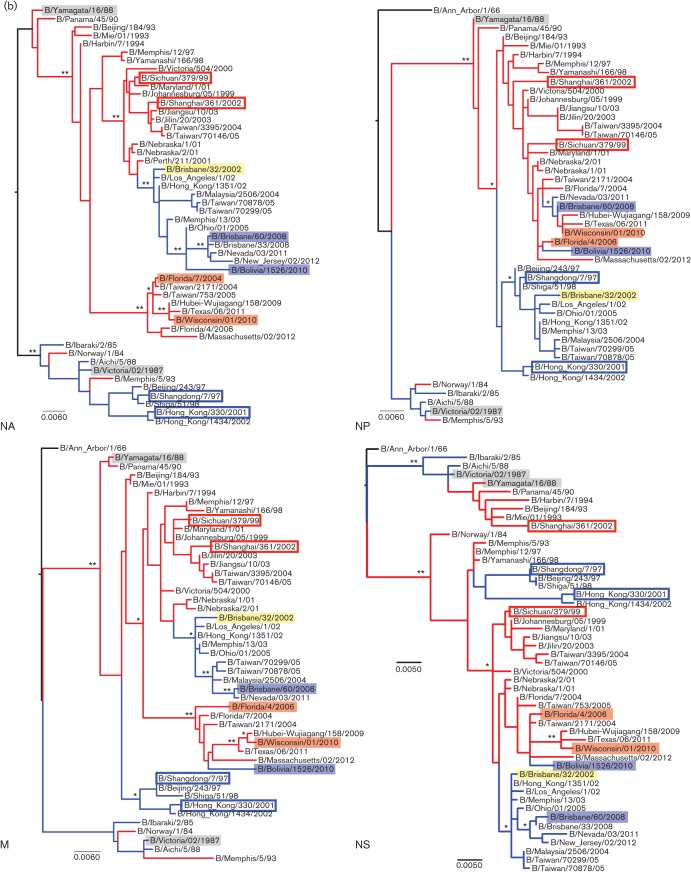
Evolutionary relationship of influenza B genes. The respective best fitting phylogenetic trees based on the nucleotide sequences of (a) PB2, PB1, PA and HA, and (b) NP, NA, M and NS segments of the viruses used this study were reconstructed by maximum-likelihood method with 1000 bootstrap replications using mega 5 software. Bars indicate the estimated frequency of nucleotide substitution per site. The blue and red shaded areas represent the B/Vic and B/Yam strains used in this study respectively. The blue and red branches annotate B/Vic and B/Yam lineage viruses according to the HA sequences respectively. The B/Vic and B/Yam prototype strains are highlighted in grey. The Bris/02 strain is highlighted in yellow. The blue and red open boxes indicate historical B/Vic and B/Yam vaccine strains specified in the text. ** and * annotate ≥95 % and ≥75 % confidence, respectively, that the branching leads to the clade classification of studied strains described in the text.

Next, we investigated the intrinsic genetic properties of each strain through phylogenetic analysis, which demonstrated the relationship of each strain to the other strains and their gene segments through time. We conducted phylogenetic analyses of all eight gene segments (PB2, PB1, PA, HA, NP, NA, M and NS) among the selected influenza B strains. As predicted from the phylogenetic analysis of the HA sequences, analysis of the HA gene arranged the strains into two main lineages where B/Bris and B/Bol viruses grouped in the B/Vic lineage and B/Fla and B/Wisc viruses in the B/Yam prototype backbone (Fig. 5a). The analysis showed the HA segments of B/Bris and B/Bol were derived from a 2002 influenza B strain [B/Brisbane/32/2002 (Bris/02)] which was isolated during the 2002–2003 influenza B infection predominant season (WHO, 2003). The strain Bris/02 was the descendant of the vaccine strain, B/Shangdong/7/1997 (Shangdong/97) but differs genetically from the vaccine strain of the same year, B/Hong Kong/330/2001 (HK/01). Similarly, the HA segments of B/Fla and B/Wisc were also derived from the period 2002–2003 [B/Shanghai/361/2002 (Shanghai/02)]. Together, recent B strains appear to be the progeny of 2002–2003 viruses after which the HA hierarchy demonstrates a temporal modification pattern. Lineage-specific trends are also observed in the polymerase segments although PA segments of the four studied influenza B viruses were shown to derive from the B/Yam lineage [B/Sichuan/379/1999 (Sichuan/99)].

The analysis of NA segments displayed a similar picture to PA, where NA segments were derived from the B/Yam lineage (Fig. 5b). Interestingly, while NA segments of B/Bris and B/Bol (and Bris/02) were descended from Sichuan/99, those of B/Fla and B/Wisc originated from a more distant B/Yam clade (sequence not available). Our analysis showed the NA segments of all studied B strains belong to the B/Yam prototype virus although from distinct clades and at least one reassortment event of PA and NA genes occurred around year 2002. However, none of these analyses complements the strain-specific infection profile observed in ferrets.

Analyses of the internal protein segments suggested further reassortment events between influenza B viruses of recent years, although all descending from B/Yam lineage, which may explain the *in vivo* differences (Fig. 5b). The analysis of NP gene segments showed the four studied viruses are closely related (clade of Sichuan/99). However, there were at least two reassortment events that resulted in a branch of B/Bris and B/Wisc and a branch of B/Fla and B/Bol. The analyses of M and NS segments display additional gene reassortment incidences that may have occurred concurrently or independently with NP reassortment events. Our results showed the M and NS segments of B/Bris strain are descended from the Bris/02 strain while those in the other three strains are grouped in a separate clade (Fig. 5b). Interestingly, our analyses also showed that the NS gene segments of influenza B evolved in a different phylogenetic pattern from that of the other segments. B/Yam and B/Vic strains share the same NS segment lineage, which descended until Shanghai/02. The NS segments in recent B viruses were differentiated from an earlier influenza B strain (B/Norway/1/84), instead of from their HA lineage ancestors (B/Yam and B/Vic). In summary, we reconstructed phylogenetic analyses of the influenza B virus segments spanning a 30-year period. We found vigorous segment reassortment occurred among influenza B strains/lineages, where the NP, M and NS gene segments showed substantial regrouping which may contribute to strain-specific *in vivo* differences in ferrets. Specifically, the genomic signature of the B/Bris virus was the most distinct among the studied viruses. Sequence differences within individual segments also accounted for the division of virus strains into subclades such as in most segments of B/Fla and B/Wisc viruses.

## Discussion

A paucity of information exists regarding the pathology of circulating influenza B viruses in humans; also there has been minimal investigation of influenza B virus pathogenesis in animal models compared to influenza A. Here we found distinct virological patterns and clinical observations from infection of influenza B strains in ferrets and suggested a genetic determinant for the clinical outcome. Differential physiological outcomes from the same lineage suggested disease severity was strain-specific and not lineage-specific. Severe diseases caused by influenza B viruses have been reported in humans, where B viruses have established lower respiratory tract infection inducing acute respiratory distress syndrome (ARDS) (Gutiérrez-Pizarraya *et al.*, 2012; Li *et al.*, 2008, 2009a) as well as other extra-pulmonary illnesses including diarrhoea and encephalitis (Chi *et al.*, 2008; Wang *et al.*, 2012). Our investigation with human influenza B virus in the ferret model demonstrated ferrets to be a useful animal model for studying the clinical and pathogenic action of influenza B infection. Importantly, we found certain influenza B strains such as B/Bris established persistent lower respiratory tract infection, which may offer a hypothesis that influenza B virus may cause moderate to severe clinical illness in humans.

Our pathological observations were in agreement with the clinical features and influenza B virus titres. We found B/Wisc induced the earliest pathology throughout the airways, correlating with early fever onset and weight loss. Although B/Bris infection did not induce early epithelial lesions, it caused the most severe small airway damage, which resembled influenza A–ARDS pathology (Gill *et al.*, 2010; Guarner & Falcón-Escobedo, 2009; Huang *et al.*, 2011). These results corresponded to the viral replication analysis where the B/Bris strain replicated at high levels throughout the ferret respiratory tract during the time-course. Interestingly, the bronchiolar and alveolar pathology in the B/Fla and B/Wisc infected animals was possibly induced by the inflammatory responses stimulated by the disseminating viral antigens or by minimal viral replication as suggested by the real-time PCR result. The biphasic weight loss during B/Bris infection may be due to broader tissue tropism exhibited by B/Bris. As the viral infection and subsequent pathology move from the upper airways and spread to the lower respiratory tract, the weight of the animal may be affected. In this scenario, the peak viral load of the upper respiratory tract may differ from that in the lower, contributing to a biphasic clinical and immunological response. This hypothesis is evidenced by the small airway pathology as it increased from day 3 to day 7 pi only in B/Bris infection. Jonsson and colleagues described another hypothesis in their model of pandemic 2009 H1N1 infection in ferrets through the use of the real-time lung imaging technique (Jonsson *et al.*, 2012). They found the biphasic weight loss might be associated with the migration of viral infection between different lobes of the lung, which may also occur during B/Bris infection when establishing lower respiratory tract infection. In our previous study, we found the kinetics of lung pathology correlated with influenza--specific antibody responses measured by haemagglutination inhibition (HI) assay (Huang *et al.*, 2011). Taking together the HI kinetics from this study (Fig. S3) and the previous studies, we found B/Bris infection induced higher HI levels on day 14 pi and broader lung pathology by day 7 pi than other influenza B infections. It is possible the persistence of lung pathology from days 3 to 7 pi caused by B/Bris virus stimulated a higher HI response at peak level. Pulmonary abnormality in B/Bol infected ferrets was minimal and correlated with absence of active viral replication in the lower lung and minimal weight loss. As suggested from previous work (Huang *et al.*, 2011, 2012; Marcelin *et al.*, 2011; Wu *et al.*, 2012), it is possible that high viral burden might not be the only attributing feature for severe clinical disease where the resulting destruction may be promoted by increased activated immune responses. However, this study demonstrated a wider distribution of efficient influenza B virus replication for B/Bris infection which reached the lower respiratory system leading to severe disease (Gill *et al.*, 2010; Harms *et al.*, 2010; Mauad *et al.*, 2010; Nakajima *et al.*, 2012; Shieh *et al.*, 2010).

Our phylogenetic analyses demonstrated an on-going complex evolution where antigenic drift of type B viruses and gene reassortment continue to take place, changing the genetic composition of circulating influenza B strains. Our phylogenetic analyses suggested that the studied B/Vic lineage viruses are progeny viruses of reassortant Bris/02 strain and the HA and NA segments are derived from Shangdon/97 (B/Vic strain) and Sichuan/99 (B/Yam strain) respectively. Furthermore, these analyses indicated several internal protein segments (PA, M and NS) in Bris/02 derived from Sichuan/99, implying these segments have probably been reassorted during the same period. The respective opposing B/Vic segments might have been replaced during early 2000 or circulated at low frequency after the dominance of HK/01-like strains. The genetic relationship of influenza B NP, M and NS may be associated with the unique behaviours of the studied strain infections *in vivo.* It has been shown that in the time period when B/Bris and B/Bris-like viruses circulated, there were unusual influenza B outbreaks and patients positive for influenza B viruses developed lower respiratory tract illness such as bronchitis and pneumonia (Esposito *et al.*, 2011; Gutiérrez-Pizarraya *et al.*, 2012). Other studies have demonstrated that the NS segments in B strains during the late 1990s were not derived from B/Yam or B/Vic but from an earlier strain isolated in 1984 (Lindstrom *et al.*, 1999; Luo *et al.*, 1999; McCullers *et al.*, 2004). Our findings present important evidence suggesting the pathogenic potential of B viruses and the potential clinical impact if a B/Bris-like virus were to emerge in a naive population.

The impact of influenza on global health and the economy is not limited to the effects of type A viruses. Although there has not been a record of an influenza B pandemic, B viruses have remained prominent and cause occasional multi-regional outbreaks. Our study demonstrated that influenza B infection with B/Bris induced moderate to severe morbidity in naive healthy male adult ferrets. It is possible that healthy adults and not only young children and the elderly are susceptible to severe influenza B infection such as the influenza B strain B/Bris (Chi *et al.*, 2008; Wang *et al.*, 2008). The rare incidence of severe disease caused by influenza B in adults may be due to adult pre-existing immunity from previous encounters with similar influenza B viruses (Paul Glezen *et al.*, 2013). The trend of increasing influenza B infection by both B/Yam and B/Vic strains around the world (Paul Glezen *et al.*, 2013) and the recent approval of influenza quadrivalent vaccines (includes B/Yam and B/Vic lineages) demonstrate the significance of influenza B viruses (WHO, 2014).

Our study provides valuable information concerning comparative clinical and pathological analyses of various influenza B strains as influenza B viruses are under-studied compared to influenza A. Our work also gives insight into the dynamics of influenza B virus genetic changes during the past three decades. These results have implications for the development of influenza B therapeutic strategies. As well, our findings showed that the B/Bris strain caused significant lower respiratory tract pathology in ferrets and insights from the genetic analysis may serve as a marker of pathogenesis during future influenza surveillance.

## Methods

### 

#### Ethics statement.

Animal work was performed in strict accordance with Canadian Council of Animal Care guidelines. University Health Network (UHN) (Toronto, Canada) has certification under the Animals for Research Act (permit numbers 0045 and 0085, Ontario Ministry of Agriculture, Food and Rural Affairs) and follows NIH guidelines (OLAW no. A5408-01). The animal use protocol was approved by the Animal Care Committee (UHN). All infections and sample collection procedures were conducted under 5 % isoflurane anaesthesia and all efforts were made to minimize pain and suffering.

#### Influenza virus and ferrets.

All influenza B viruses (B/Brisbane/60/2008, B/Florida/04/2006, B/Wisconsin/01/2010 and B/Bolivia/1526/2010) were provided in embryonic chicken egg allantoic fluid aliquots by the Influenza Reagent Resource, Influenza Division, WHO Collaborating Center for Surveillance, Epidemiology and Control of Influenza, Centers for Disease Control and Prevention, Atlanta, GA, USA. Male ferrets 4–6 months old were bred in an on-site specific pathogen free ferret colony (UHN). Ferrets were shown to be seronegative by HI assay against circulating influenza A and B strains using the 2010–2011 WHO Influenza Reagent kit for identification of influenza isolates. Ferrets used in this study were also naive to the four virus strains used here.

#### Ferret infection and monitoring.

Maintenance and monitoring of ferrets upon infection followed previously published protocols (Huang *et al.*, 2011). Ferrets were randomly selected and pair-housed in cages contained in bioclean portable laminar-flow clean-room enclosures (Lab Products) in a biosafety level 2 facility (BSL-2). Inactivity level, nasal discharge, sneezing, body temperature [measured with subcutaneous implantable temperature transponder (BioMedic Data Systems)] and weight of animals were measured on day 0 prior to infection and each day thereafter. For infection, each ferret was anaesthetized and infected intranasally with 1 ml inoculum (0.5 ml to each nostril) at the dosage of 10^6^ 50 % egg infectious dosage (EID_50_). Viruses were diluted in saline buffer before infection. All virus preparation work was conducted in a BSL-2 facility. Nasal discharge, prominence, type, colour, and location of crusty nose, mucus and exudates/fluids were recorded. Scores were calculated daily and peak values for each infection are summarized (Fig. 1, panel c). The inactivity index of each viral infection was calculated using the scores observed daily. The scoring protocol was adapted from that described by Reuman *et al.* (1989): 0, alert and playful; 0.5, alert but playful only when given incentives; 1, alert but not playful when given incentives; 2, neither alert nor playful even when given incentives. To calculate the index, a value of 1 was first added to each daily score of each animal followed by the summation of all the values from all infected animals from day 1 to 7 pi. The total number was divided by the total observation incidence (i.e. the total number of animals multiplied by 7 days), rendering the index value: 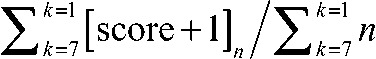, where *n* equals the total number of observations from day 1 to 7 pi. The value of 1 added to each observation score resulted in a minimum value of 1.000 as an inactivity index for each infection.

#### Viral load.

The procedure was as previously described (Huang *et al.*, 2012). Viral loads in the upper, middle and lower respiratory tract were determined by end point titration of nasal washes or respiratory tissue homogenates on MDCK cells followed by haemagglutination of turkey erythrocytes (Lampire Biological Laboratories) with MDCK supernatant to calculate 50 % TCID_50_. Nasal washes from infected ferrets were collected using 1 ml of nasal wash buffer (1 % BSA, 100 U penicillin ml^−1^, 100 µg streptomycin ml^−1^ in PBS). Tracheas and lungs were collected on 3 and 7 days pi from euthanized ferrets. Nasal washes and tissue homogenates were titrated in vDMEM [Dulbecco's modified Eagle’s medium (DMEM) containing 1 % BSA, 25 mM glucose, 1 mM sodium pyruvate, 4 mM glutamine, 100 U penicillin ml^−1^, 100 µg streptomycin ml^−1^, 50 µg gentamicin ml^−1^ and 1 µg TPCK-trypsin ml^−1^] and incubated on MDCK cells at 37 °C, 5 % CO_2_. After 2 h, supernatant was aspirated and replaced with fresh vDMEM followed by 6 days at 37 °C, 5 % CO_2_. Supernatant was examined for presence of virus by haemagglutination of turkey erythrocytes. The viral titres were determined as the reciprocal of the dilution resulting in 50 % HA positivity as log_10_ (TCID_50_ ml^−1^). All cell culture reagents were obtained from Invitrogen except for TPCK-trypsin (Sigma-Aldrich) and BSA (Wisent).

#### Histopathology.

Tissue samples were formalin-fixed after harvesting from euthanized ferrets. Formalin was used to perfuse lung samples. Tissues were embedded with paraffin wax and sectioned. Haematoxylin and eosin staining was performed for histopathological assessment. Mock-infected male (age-matched) ferret tissues were derived from animals treated with PBS intranasally (Huang *et al.*, 2011).

#### RNA extraction and sequencing of B/Bolivia/1526/2010.

Total RNA was extracted from chicken egg allantoic fluid stock of B/Bolivia/1526/2010 using the QIAamp Viral RNA Mini kit according to the manufacturer’s protocol (Qiagen). A sequencing library was prepared using the Truseq RNA Prep kit V2 (Illumina) according to the manufacturer’s instructions. Single end, 50 bp sequencing was performed in an Illumina HiSeq2500 at the Donnelly Sequencing Centre of the University of Toronto (http://dsc.utoronto.ca/dsc/index.html). To extract viral sequences, the resulting short-reads were aligned to the genomic segments of the closely related B/Brisbane/60/2008 (Table S1) using the DNA aligner Bowtie2 program v2.0.6 (Langmead & Salzberg, 2012). Finally, the consensus sequences were generated with the Samtools program v0.1.12 (Li *et al.*, 2009b). 

#### Phylogenetic analyses.

Phylogenetic analyses of all nucleotide sequences were conducted in mega v6.0.2 software (Tamura *et al.*, 2011) using maximum-likelihood method based on the Tamura–Nei model (Tamura & Nei, 1993) with bootstrap replications (*n* = 1000) to determine the best-fitting tree for each segment. Table S1 summarizes the accession numbers of the sequences obtained from GISAID or GenBank (http://www.ncbi.nlm.nih.gov/nuccore), labelled with *.

#### Statistics.

One-way analysis of variance (ANOVA) was conducted to compare the temperature and weight change profiles during the four strain infections in Fig. 1 and viral burden kinetics in Fig. 2. If statistically significant difference (*p*<0.05) was found on a particular day pi, Bonferroni–Holm analysis (post-hoc analysis) was then applied between the results of B/Bris infection and the other three infections in a pairwise manner.
